# Assessment of the sensitivity of thoracic injury criteria to subject-specific characteristics using human body models

**DOI:** 10.3389/fbioe.2023.1106554

**Published:** 2023-02-13

**Authors:** Ana Piqueras, Johan Iraeus, Bengt Pipkorn, Francisco J. López-Valdés

**Affiliations:** ^1^ Department of Mechanical Engineering, EINA, University of Zaragoza, Zaragoza, Spain; ^2^ Division of Vehicle Safety, Department of Mechanics and Maritime Sciences, Chalmers University of Technology, Gothenburg, Sweden; ^3^ Autoliv Research, Vargarda, Sweden; ^4^ Instituto de Investigación Tecnológica (IIT), Department of Mechanical Engineering, ICAI, Universidad Pontificia Comillas, Madrid, Spain

**Keywords:** human body model (HBM), injury metrics, nearside, oblique impact, thoracic injury risk, personification

## Abstract

**Introduction:** Chest deformation has been proposed as the best predictor of thoracic injury risk in frontal impacts. Finite Element Human Body Models (FE-HBM) can enhance the results obtained in physical crash tests with Anthropometric Test Devices (ATD) since they can be exposed to omnidirectional impacts and their geometry can be modified to reflect specific population groups. This study aims to assess the sensitivity of two thoracic injury risk criteria (PC Score and Cmax) to several personalization techniques of FE-HBMs.

**Methods:** Three 30° nearside oblique sled tests were reproduced using the SAFER HBM v8 and three personalization techniques were applied to this model to evaluate the influence on the risk of thoracic injuries. First, the overall mass of the model was adjusted to represent the weight of the subjects. Second, the model anthropometry and mass were modified to represent the characteristics of the post-mortem human subjects (PMHS). Finally, the spine alignment of the model was adapted to the PMHS posture at *t* = 0 ms, to conform to the angles between spinal landmarks measured in the PMHS. The following two metrics were used to predict three or more fractured ribs (AIS3+) of the SAFER HBM v8 and the effect of personalization techniques: the maximum posterior displacement of any studied chest point (Cmax), and the sum of the upper and lower deformation of selected rib points (PC score).

**Results:** Despite having led to statistically significant differences in the probability of AIS3+ calculations, the mass-scaled and morphed version provided, in general, lower values for injury risk than the baseline model and the postured version being the latter, which exhibited the better approximation to the PMHS tests in terms of probability of injury. Additionally, this study found that the prediction of AIS3+ chest injuries based on PC Score resulted in higher probability values than the prediction based on Cmax for the loading conditions and personalization techniques analyzed within this study.

**Discussion:** This study could demonstrate that the personalization techniques do not lead to linear trends when they are used in combination. Furthermore, the results included here suggest that these two criteria will result in significantly different predictions if the chest is loaded more asymmetrically.

## 1 Introduction

Anthropometric Test Devices (ATD), also known as crash test dummies, are commonly used in regulatory procedures and consumer testing programs in the assessment of the potential injury risks of car occupants (IIHS 2012; NHTSA 2015; EuroNCAP 2018; ANCAP 2020). This assessment is based on the use of injury criteria, which relate the value of a measured physical magnitude (such as force or deformation) with a certain probability (p) of sustaining an injury. In the case of the thorax, several injury criteria have been used historically to predict the probability of chest injuries (Kroell et al., 1974; Lau and Viano 1986; Kleinberger et al., 1998). Contemporary research associated with the development of the Test Device for Human Occupant Restraint (THOR) ATD has proposed injury criteria based on the 3D measurement of chest deformation as the best predictor of the risk of thoracic injuries in frontal impacts (Davidsson et al., 2014; Poplin et al., 2017).

Poplin et al. (2017) developed a multipoint chest deformation injury criterion based on an accelerated failure time model from experimental data obtained from a set of 45 post-mortem human subjects (PMHS) exposed to 13 different impact conditions using age as a covariant. After reproducing the same impact conditions with the THOR ATD, Poplin et al. (2017) proposed the total and differential chest deformations (PC Score) and Cmax as the most reliable injury criteria to predict the probability of thoracic injuries in frontal impacts. The authors observed that the predicted probability of AIS3+ (three or more fractured ribs (AAAM 2015)) thoracic injuries obtained by either injury criteria was very similar. The same observation was reported in Lopez-Valdes et al. (2018), which used both injury criteria with the THOR dummy to predict the thoracic injuries of three elderly male PMHS in sled frontal impacts. This study also highlighted that both criteria had underestimated the actual risk of thoracic injury observed in the PMHS tests.

Studies focusing on oblique impacts have suggested that oblique loading can cause larger chest deformations than frontal loading (Acosta et al., 2016; Piqueras et al., 2022). However, current ATD were developed for evaluation of either frontal impacts (Hybrid III, THOR) or side impacts (EuroSID, WorlSID) and, thus, the response under oblique impact may not be biofidelic. The use of detailed Finite Element Human Body Models (FE-HBM) can enhance the information obtained from physical crash tests with crash test dummies as FE-HBM can be exposed to omnidirectional impacts. Additionally, the FE-HBM description of the material properties of the tissue allows, at least theoretically, the calculation of injury risk to be based on strain measurements, a magnitude that is more likely to be related to the actual mechanisms causing the tissue to fail. Accordingly, several studies have proposed injury criteria for FE-HBM based on strain (Laituri et al., 2005; Forman et al., 2012; Iraeus et al., 2020). Two main groups can be distinguished: deterministic and probabilistic criteria. In the former, the strain predicted by the FE-HBM is compared to a previously accepted injury threshold and if the strain exceeds the threshold, an injury is predicted. In the latter, the predicted strain is transformed into the probability of sustaining such strain given the known distribution of strain in the population (that needs to be known/estimated before). Several studies using deterministic methods have shown that these methods are less sensitive to changes in the restraint conditions than other injury criteria not based on strain (Song et al., 2011; Larsson et al., 2019). Forman et al. (2012) was the first study developing a probabilistic injury criteria approach for FE-HBM and Pipkorn et al. (2019) showed that this method was capable of predicting the number of fractured ribs observed in PMHS sled tests. However, to succeed in injury risk prediction, the FE-HBM had to be developed to accurately predict the actual strain of the tissue. Therefore, the injury risk functions are dependent on model characteristics such as the mesh size of each FE-HBM and have to be developed and validated for each loading scenario (Forman et al., 2022), which is not always feasible.

Thus, in parallel to strain-based thoracic injury criteria, several studies have used HBM chest deformations as a potential predictor of thoracic injuries, similar to what is done with ATD. For instance, Mendoza-Vazquez et al. (2015) developed a set of AIS2+ [two fractured ribs (AAAM 2015)] thoracic injury risk curves using several deformation-based criteria such as Dmax, Cmax, VCmax, and DcTHOR, the latter a multi-point chest deflection metric proposed by Davidsson et al. (2014). The study concluded that DcTHOR resulted in the best predictor for the thoracic injury risk, despite being a metric developed to be used with ATD. In addition, Mendoza-Vazquez et al. (2015) also found that injury metrics based on multi-point measurements (DcTHOR and Cmax) were less sensitive to variations in the material properties of the FE-HBM, making these metrics particularly suitable to be used with FE models. Current studies show multiple examples of the application of deformation-based criteria to the prediction of chest injuries using FE-HBM such as Brolin and Wass (2016), which used DcTHOR and Dmax metrics with the THUMS model for the assessment of the injury protection provided by a safety-vest in equestrian riders, or Grébonval et al. (2021), which used the Cmax and PC Score to compare the thoracic injury risk predicted by the GHBMC HBM and the THOR ATD in frontal impacts in reclined occupant positions.

One of the advantages of FE-HBM is the possibility of modifying the geometry of the model to represent specific groups of people within the population. This possibility has resulted in the development of parametric models capable of reproducing subject-specific characteristics (Shi et al., 2014; Hwang et al., 2016; Hu et al., 2017). Another level of personalization of FE-HBM focused on the possibility of mimicking specific initial postures of occupants (or PMHS if cadaveric tests are being used to benchmark the models). Poulard et al. (2015) found that the HBM pre-impact posture altered the predicted kinematics and rib fracture risk in frontal impacts, even if it had a limited effect on the amplitude of the outputs. However, it is not entirely clear yet how these levels of personalization contribute to the accuracy of FE-HBM thoracic injury risk predictions. Previous research has shown that the modification of the anthropometry and pre-impact posture of FE-HBM contributed to improving the HBM predictions of the external occupant kinematics (Piqueras et al., 2018; Larsson et al., 2019). Despite this improvement, the rib deformation patterns were not correctly captured by the personalized models, and the chest deflection measured in the reference PMHS tests was underpredicted (Piqueras et al., 2022).

Thus, the assessment of the effect of the personalization techniques on the injury risk prediction of FE-HBM requires additional research. This study aims to assess the sensitivity of two multi-point deflection metrics commonly used with FE-HBM to estimate the risk of thoracic injuries (i.e., Cmax and PC Score) to several personalization techniques in nearside oblique impacts.

## 2 Materials and methods

### 2.1 Reference physical tests

In order to assess the sensitivity of the PC Score and Cmax metrics under oblique loading, three 30° nearside oblique PMHS sled tests (A, B, and C, see Table 1) were chosen as reference data for the subject-specific characteristics applied on the HBM modifications (López-Valdés et al., 2016). These PMHS tests have been described in detail in several other publications and are used here only as the physical reference case to build the environment and initial conditions of the different FE models under study (López-Valdés et al., 2016; Piqueras et al., 2018; Piqueras et al., 2022). In the physical tests, the occupants were restrained with two different versions of a passenger-side three-point seatbelt. In restraint system version 1 (RSv1, used with PMHS A and B), the shoulder belt was pre-tensioned at 2 kN and included a force-limiter at 4.5 kN, and the lap belt was equipped with a 3.5 kN pre-tensioner. In RSv2 (used PMHS C), the same restraint system was used but the shoulder pre-tensioner was not activated in the test. The physical tests were carried out using a modified version of the Gold Standard fixture which was fully described in Pipkorn et al. (2016). The three subjects, A, B, and C, sustained AIS3 thoracic injuries.

**TABLE 1:** Test setup and PMHS information including injury outcome.

	Restraint system v1		Restraint system v2
PMHS	PMHS A	PMHS B	PMHS C
Impact angle (deg)	30		
Velocity (km/h)	35		
Seatbelt	3-point		
Pretensioner	Shoulder (2 kN)		Lap belt 3.5 kN
	Lap belt 3.5 kN		
Force Limiter	Shoulder belt 4.5 kN		
Configuration	Passenger		
Age	66	68	60
Sex	Male	Male	Male
Stature (cm)	175	169	170.5
Weight (kg)	47	53	57
Fractured ribs (total number of fractures)	15 (22)	5 (7)	10 (11)

The sled acceleration pulse, obtained from the physical tests, was applied to the sled FE models to mimic the mechanical sled loading conditions. The pre-tensioner and force limiter triggering times and loading curves were adjusted to accurately represent the time-history seat-belt forces acquired from the physical tests. The sled fixture and belt models used for this study were validated in Pipkorn et al. (2016). The coordinates for the D-ring, footrest, buckle, and attachments, were adjusted in the simulation environment taking as reference the location used during the physical tests (López-Valdés et al., 2016). Additionally, the belt was routed over the HBM chest using the acquired position of the belt positioning markers at t = 0 ms.

### 2.2 Human body model and personalization techniques

The SAFER HBM v8, which is based on the commercial THUMS v3 (Iwamoto et al., 2002), was used for this study. The modifications of SAFER HBM v8 included an updated ribcage (Iraeus et al., 2017) and a new definition of the lumbar spine mechanical properties (Afewerki 2016). The trabecular bone of the sternum and ribs was modeled with hexahedral elements as well as the mid-substance of the costal cartilage. Cortical bone and the perichondrium layer were modeled using quadratic shell elements. A piecewise linear plasticity material was used in the formulation of the material properties of the chest elements. Rib material properties were based on experimental data obtained from rib coupon tests in a sample including individuals between 18 and 81 years old (Kemper et al., 2005; Kemper et al., 2007).

The personalization of the SAFER HBM v8 model to the PMHS exposed to the oblique impacts was carried out in three steps.

**FIGURE 1:**
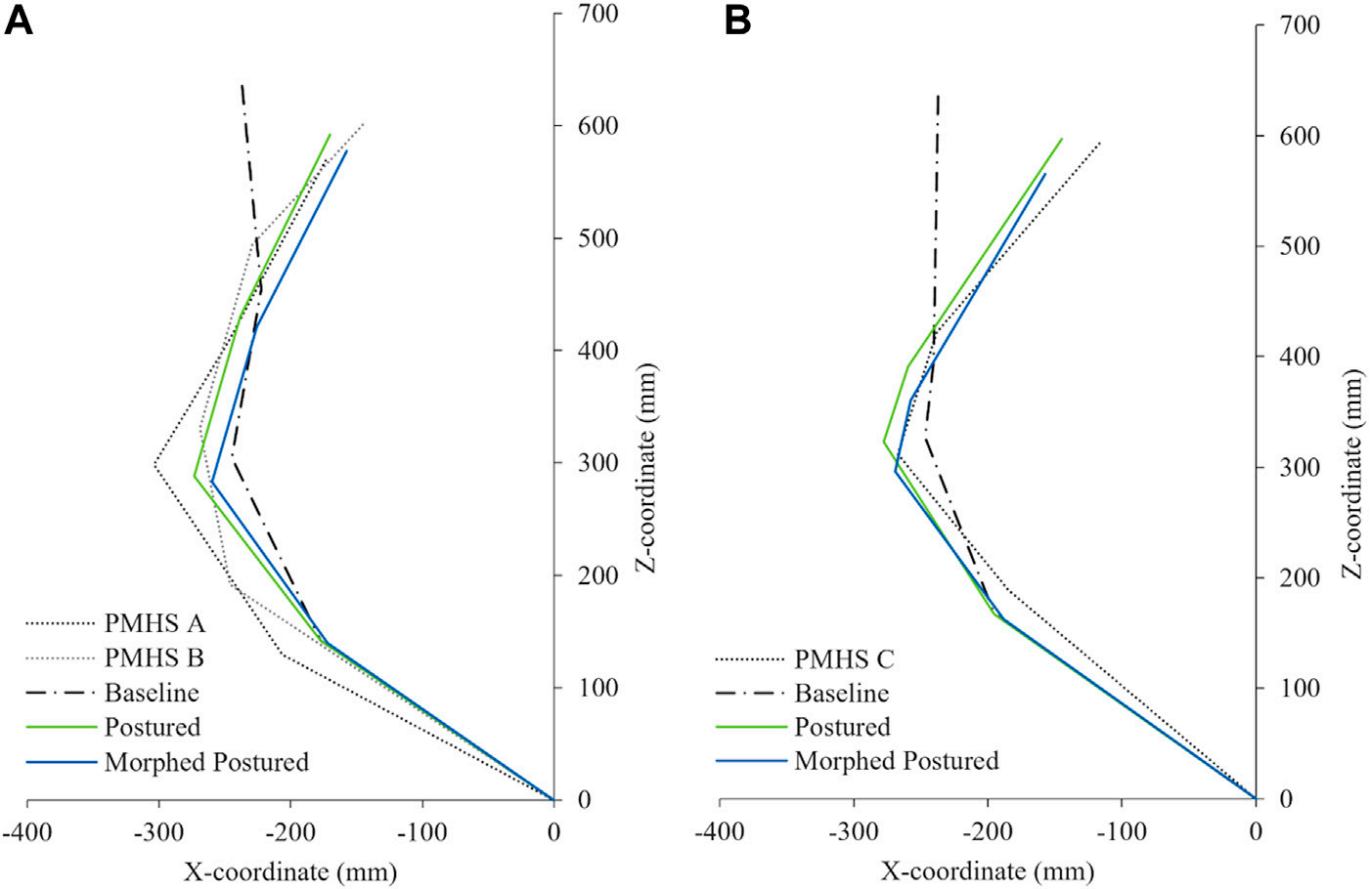
Spine alignment representation at *t* = 0 ms of the PMHS, THUMS baseline model, and postured models. **(A)** RSv1 and **(B)** RSv2.

1. First, the overall mass of the model was adjusted to represent the weight of the subjects preserving the external shape and size of the baseline model. The density of the fat and flesh parts was modified to accomplish the PMHS mass from the THUMS original weight (see Table 2).
2. Second, the model anthropometry and mass of the baseline model were modified to represent the characteristics of the PMHS used in the tests described in López-Valdés et al. (2016) by means of the Kriging interpolation module included in the PIPER software v1.0.0. The anthropometric dimensions (lengths and contours of the body parts) used as targets for morphing, were extracted from the seated anthropometry measurements taken before the tests (see Supplementary Table 1-1).
3. Finally, the influence of the initial posture in the prediction of chest injuries was analyzed by aligning the spine curvature of the FE-HBM to the actual spine curvature of the PMHS measured at t = 0 ms (see Figure 1). This was performed using an independent pre-simulation where a prescribed motion was applied to the head, T1, T8, L2, and H-Point, to conform to the relative angles between landmarks measured on the PMHS (see Supplementary Tables 1-2, 1-3). Due to the specifics of the PMHS preparation process, the clusters for subject C (RSv2), were attached to T4, T7, and L1, thus, the spine alignment was done using these landmarks in this case.

**TABLE 2:** Mass modification of the mass-scaled models’ flesh and fat parts.

Restraint system v1					
	PMHS A	PMHS B	Unscaled SAFER HBM v8 model	Density scale factor	Mass-scaled models
Fixed mass parts (skeleton, internal organs, etc.) (kg)	—	—	25.3	1	25.3
Fat and flesh parts (kg)	—	—	52.29	0.472	24.7
Total mass (kg)	47	53	77.59	—	50

Restraint system v2					
	PMHS C		Unscaled SAFER HBM v8 model	Density scale factor	Mass-scaled models
Fixed mass parts (skeleton, internal organs, etc.) (kg)	—		25.3	1	25.3
Fat and flesh parts (kg)	—		52.29	0.606	31.7
Total mass (kg)	57		77.59	—	57

These three modifications were applied in different combinations to the baseline FE-HBM model resulting in six different versions of the HBM for each restrain configuration.

(1) Baseline model: the SAFER HBM v8 model remains unmodified representing a 50th percentile male occupant.
(2) Baseline postured model: The spine alignment of the baseline model was adjusted to the PMHS initial position.
(3) Scaled mass model: The density of the outer flesh parts was adjusted until the full model mass reached the overall weight of the subject.
(4) Scaled mass postured model: In this version, the spine of the scaled mass model was adapted to the initial posture of the PMHS.
(5) Morphed model: The geometry and the mass of the baseline SAFER HBM v8 model were modified to represent the PMHS anthropometry using the PIPER software.
(6) Morphed postured model: The mass, the geometry, and the posture were modified to represent the characteristics of the subject.

The targeted mass, anthropometry, and spine alignment have been calculated considering the subjects tested with each RSv. The anthropometry of PMHS A and PMHS B was averaged to develop a single personalized HBM. This was decided due to the large similarities in anthropometry (see Supplementary Table 1-1) and initial posture (see Supplementary Tables 1-2, 1-3) of the two subjects that would have resulted in minimal differences in the corresponding HBM. Separately, data from PMHS C was used to develop the HBM versions for the RSv2. Thus, a total of 12 simulations have been carried out, corresponding to the six mentioned versions for each of the two RSv (see Table 3).

**TABLE 3:** Model versions and modifications applied.

Model Number	Model name	Mass scaling	Morphing	Posturing
(1)	Baseline			
(2)	Baseline postured			Yes
(3)	Scaled mass	Yes		
(4)	Scaled mass postured	Yes		Yes
(5)	Morphed	Yes	Yes	
(6)	Morphed postured	Yes	Yes	Yes

### 2.3 Chest deflection measurement

**FIGURE 2:**
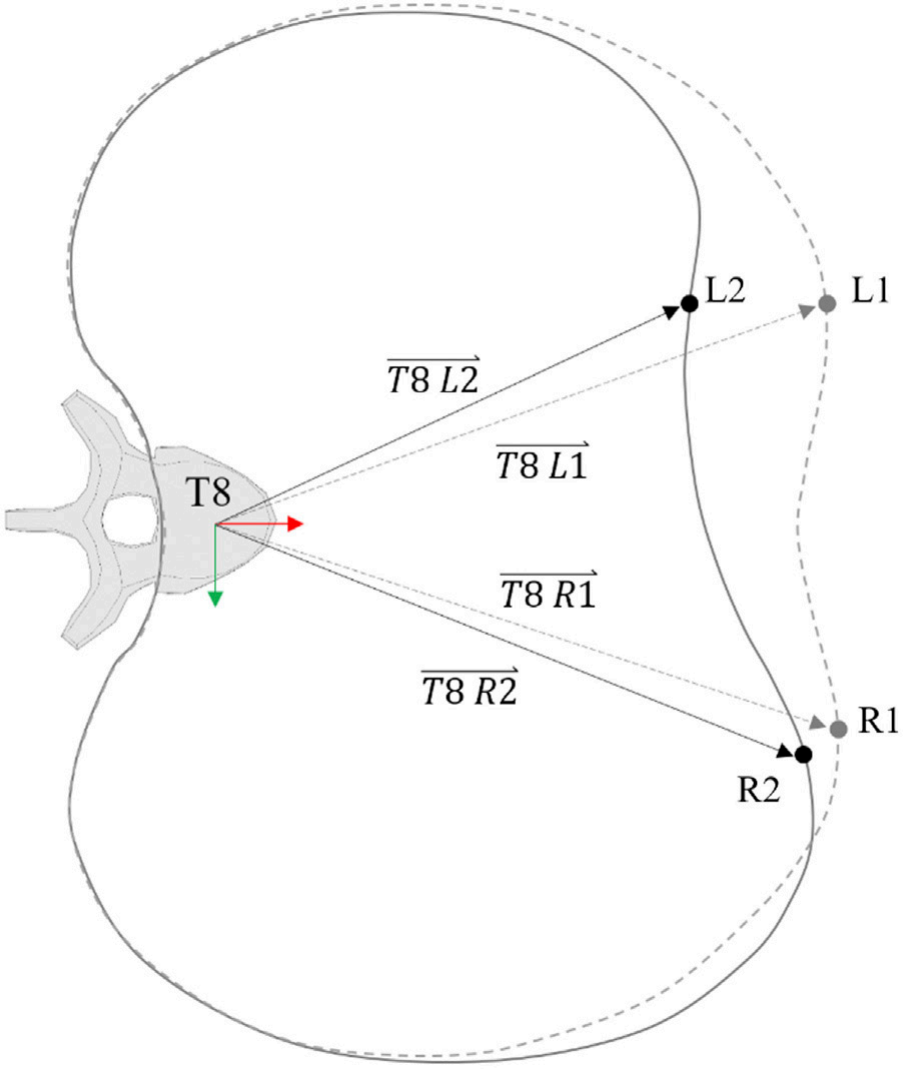
Chest deflection representation along one rib level. The slashed line represents the undeformed rib profile.

Chest deflection was calculated at the 4^th^ and 8^th^ ribs bilaterally (UL, UR, LL, and LR). The deflection was calculated as the change in length of a vector joining the location of a marker cluster that was used to define a local coordinate system (LCS) rigidly attached to the rib points mentioned above and the origin of an LCS attached to the eighth thoracic vertebra (T8) ( $\left|\vec{T8\;L1}\right|-\left|\vec{T8\;L2}\right|$ , see Figure 2) (Piqueras et al., 2022). The relative distances of the marker clusters from the sternum along each rib (see Table 4) were used to select analogous points on the different versions of the SAFER HBM so that the calculation of the deflection would be consistent across models.

**TABLE 4:** Relative position of the nodes used for chest deflection measurement from the sternum along each rib for the three subjects.

Subject	PMHS A		PMHS B		PMHS C	
Aspect	Right	Left	Right	Left	Right	Left
4th rib	32.6%	15.5%	24.2%	26.4%	25.7%	31.4%
8th rib	39.5%	42.3%	38.5%	41.9%	40.5%	39.5%

The differences in the position of the upper chest landmarks between PMHS A and B in the experiments were large. To avoid influencing the results by averaging the position of the clusters, the chest deflection of the RSv1 model versions was analyzed regarding both landmark locations (PMHS A and B) resulting in 18 comparisons (models A, B, and C and versions 1 to 6). 

### 2.4 Injury predictors

Two main predictors based on multi-point chest deformation measurements were considered: Cmax and PC Score. Cmax computes the maximum posterior resultant displacement of any studied rib point of the chest, independently of the displacement of the rest of the rib points. For instance, as shown in Figure 2, Cmax will compute the deflection of the left point (L), regardless of the right point (R) deflection. PC Score computes the sum of the maximum deformation measured at the upper and lower chest (UPtot and LOWtot) and the maximum differential deformation of the upper and lower rib measurement points (UPdif and LOWdif) (Poplin et al., 2017). Returning to the example, PC Score will compute, not only the maximum deformation that occurred at L and R points but also the differential deformation between these two points for the upper and lower chest.

In order to consistently compare the results obtained from both injury criteria, the probability of sustaining an AIS3+ injury to the chest (AAAM 2015) was calculated according to the injury risk functions developed by Poplin et al. (2017). This formulation uses age as the co-variant for the calculation, thus, the age of the three subjects at the time of death was considered (66, 68, and 60 y. o. for models A, B, and C respectively).

### 2.5 Statistical analysis

First, the equivalence of using Cmax or PC Score for the p (AIS3+) calculation was assessed. Due to the reduced number of samples, a non-normal distribution has been assumed. Thus, a non-parametric Wilcoxon signed-rank test for paired samples was carried out for the analysis. The results of p (AIS3+) calculated based on Cmax for all the model versions were compared with those calculated based on PC Score, establishing equal medians as a null hypothesis (H). 

Second, the influence of the three personalization techniques (mass scaling, morphing, and posturing) on the p (AIS3+) was analyzed by comparing the results of the different model versions. The simulation results were separated into two groups for each personalization technique: Group one (reference group), in which the model did not include the monitored personalization technique, and group two, with the models that had been modified including that technique (see columns in Table 3). The grouping for the analysis of each modification is shown in Table 5.

**TABLE 5:** Model versions groups for the analysis of the influence of the three personalization techniques.

Personalization technique	Group 1	Group 2
Mass scaling		(3) Scaled mass
	(1) Baseline	(4) Scaled mass postured
	(2) Baseline postured	(5) Morphed
		(6) Morphed postured
Morphing	(1) Baseline	
	(2) Baseline postured	(5) Morphed
	(3) Scaled mass	(6) Morphed postured
	(4) Scaled mass postured	
Posturing	(1) Baseline	(2) Baseline postured
	(3) Scaled mass	(4) Scaled mass postured
	(5) Morphed	(6) Morphed postured

Then, the results of p (AIS3+) were compared using a non-parametric Mann-Whitney *U* test for non-paired samples, assuming equal means as a null hypothesis. This comparison was done separately for the PC Score-based calculation and the Cmax-based calculation resulting in a total of six analyses (3personalization techniques x 2 metrics).

In both analyses, the significance level of the statistical tests was established at a *p*-value of <0.05.

## 3 Results

The maximum deflection results from the PMHS tests were extracted from Piqueras et al. (2022) and are summarized in Supplementary Figures 1-1. Maximum deflection values for the four rib landmarks obtained from the model versions are exposed in Supplementary Figures 1-2–1-4; Supplementary Table 1-4.

### 3.1 Injury risk by Cmax and PC score

The results of p (AIS3+) computed using Cmax and PC Score obtained for each HBM version are shown in Table 6.

**TABLE 6:** Probability of AIS3+ for the different model versions based on the Cmax and PC Score metrics.

	p(AIS3+|Cmax, age)			p(AIS3+|PC score, age)		
	RSv1		RSv2	RSv1		RSv2
Model	A (%)	B (%)	C (%)	A (%)	B (%)	C (%)
(1) Baseline	43.00	38.48	35.64	66.83	56.99	51.39
(2) Postured	51.99	58.48	38.08	76.08	62.55	50.96
(3) Mass scaled	47.70	33.93	41.08	45.97	37.07	41.25
(4) Mass scaled postured	26.55	30.60	29.92	39.16	29.68	40.17
(5) Morphed	40.79	24.91	18.09	45.59	32.77	18.93
(6) Morphed postured	21.61	18.48	11.23	40.15	28.80	18.24

According to the data observed in Table 6, the postured HBM version (2) obtained, in general, the highest values of injury risk with either PC Score-based or Cmax-based calculations, while the morphed postured version (6) exhibited the lowest probability of injury risk.

### 3.2 Analysis of the influence on the use of Cmax and PC score

**FIGURE 3:**
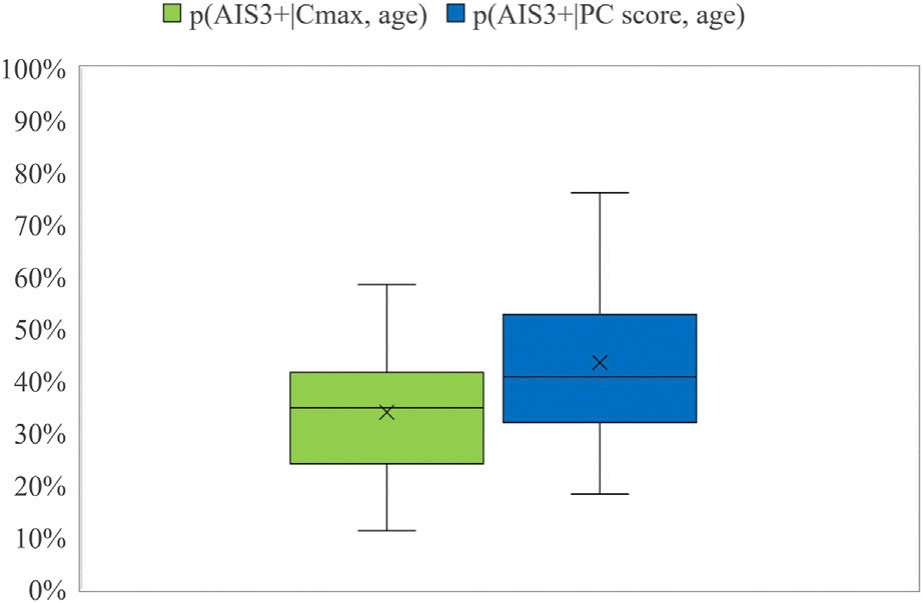
Box plot for the probability of AIS3+ calculated using Cmax and PC Score.

According to the data, the p (AIS3+) calculated based on the PC Score resulted in higher probability values than those based on the Cmax metric, as can be seen in Figure 3.

This hypothesis was confirmed by the Wilcoxon test, obtaining a *p*-value of 0.0007, and even when each RS was analyzed separately, obtaining a *p*-value of 0.005 for RSv1 and a *p*-value of 0.03 for Rsv2. However, these differences were found to be higher for the RSv1 than for the RSv2.

### 3.3 Analysis of the influence of the personalization techniques

In order to evaluate the effect of each technique, the results shown in Table 6 were separated into two groups according to what has been shown in Table 5. The resulting *p*-values for the Mann-Whitney U tests are shown in Table 7.

**TABLE 7:** Summary of the *p*-value of the p (AIS3+) discretized by personalization technique and injury metric (*α* = 0.05).

	Mass scaling	Morphing	Posturing
*p*-value based on p (AIS3+|Cmax, age)	0.016	0.007	0.354
*p*-value based on p (AIS3+|PC Score, age)	0.001	0.011	0.566

This analysis revealed that the mass scaling and morphing of the HBM significantly influenced the prediction of AIS3+ while the posturing did not show statistically significant differences in the results, regardless of the injury metric used.

## 4 Discussion

In the present work, the ATD deformation-based criteria, PC Score and Cmax, have been applied to the SAFER HBM v8 under nearside oblique impacts to evaluate the influence of several proposed personalization techniques.

Poplin et al. (2017) developed the formulation of the injury risk functions associated with the PC Score and Cmax based on a set of 45 PMHS tests under 13 different test conditions, considering the age of the subject as a covariant of the injury risk calculation. The results of that study showed that both deformation-based criteria produced similar results in terms of injury prediction. A later study assessing the prediction capability of thoracic injuries of the THOR ATD found the same similarities between the two injury criteria (Lopez-Valdes et al., 2018). These two studies were focused on frontal impact configurations, and similar results can be possibly explained because the study by Poplin et al. (2017) included only one oblique impact in the formulation of the injury risk functions. On the contrary, the present study found differences between the two criteria, revealing that the calculated probability of sustaining three or more rib fractures based on the PC Score showed significantly higher values (*p* < 0.05) than those based on the Cmax metric. These results suggest that these two formulations can result in a different risk estimation in impact configurations that cause a more asymmetric deformation of the chest, such as in the case of the oblique impacts considered here. The formulation of the PC Score includes weighting coefficients assigned to the terms in the formula (UPtot, LOWtot, UPdif, and LOWdif). The UPdif and LOWdif components resulted in the highest weighting coefficients in the formulation. Thus, the more influential parameters on the PC Score calculation are strongly related to the differential deformations as a relevant injury mechanism. Oblique loading has been shown to produce larger chest deformations than frontal impacts (Acosta et al., 2016; Piqueras et al., 2022), leading to more asymmetric chest deformations and, thus, to higher values of the LOWdif and UPdif components. Since Cmax does not consider the differential deformations, the probability of injury calculated based on this criterion showed lower values of injury risk under the oblique impact configuration. This can also be observed for the nearside oblique test condition, in particular, analyzed by Poplin et al. (2017) in which the p (AIS3+) for the nearside oblique configuration was found to be 6.05 percent points higher when the PC Score was applied compared with the same calculation based on Cmax, consistent with the findings of this work. The summary of results including the deflection values at each thoracic landmark and the values of the injury metrics are available in Supplementary Table 1-4.

In a previous study of the THOR injury prediction capability using the same PMHS sled tests as was used in the present study as a reference, the calculations based on the ATD thorax deformation showed a 31% of probability of sustaining three or more rib fractures using Cmax as deformation-based criteria for a 65-year-old occupant (Pipkorn et al., 2016). This value is lower than the prediction obtained with the baseline HBM in this study using the same deformation metric (39.04% ± 3.71) and, therefore, also lower than the values obtained using PC Score-based calculation (58.4% ± 7.82). These differences can be attributed to the lack of X-deflection (positive) of the lower right chest of the THOR ATD, something that occurred in the corresponding PMHS sled tests (please see Supplementary Figures 1-1–1-4) (Piqueras et al., 2022).

As mentioned in the introduction, a potential advantage of the use of HBMs is that they allow the study of strain-based injury criteria. In these methods, the strain at any point of the ribcage can be computed and related to the number and location of the fractures when compared to PMHS tests. However, these methods are dependent not only on the HBM biofidelity but also on model construction characteristics such as mesh size. This means that to obtain comparable results, the local strain injury risk functions have to be developed and validated for each specific loading scenario across diverse HBMs (Forman et al., 2022). For this reason, strain-based criteria were not used for the present study.

**FIGURE 4:**
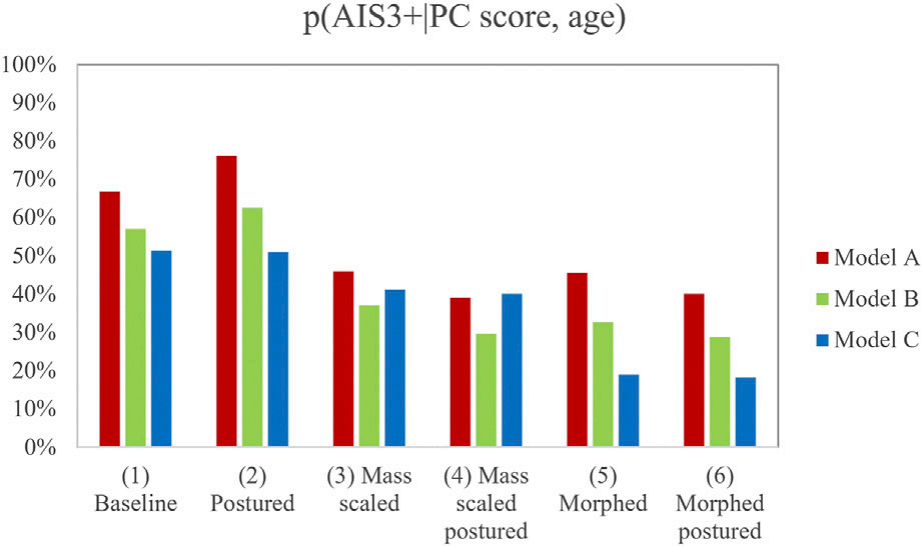
Probability of AIS3+ for the different model versions based on the PC Score metric. Note that there are 18 results to account for the differences in the position of the upper chest landmarks between PMHS A and B in the experiments.

**FIGURE 5:**
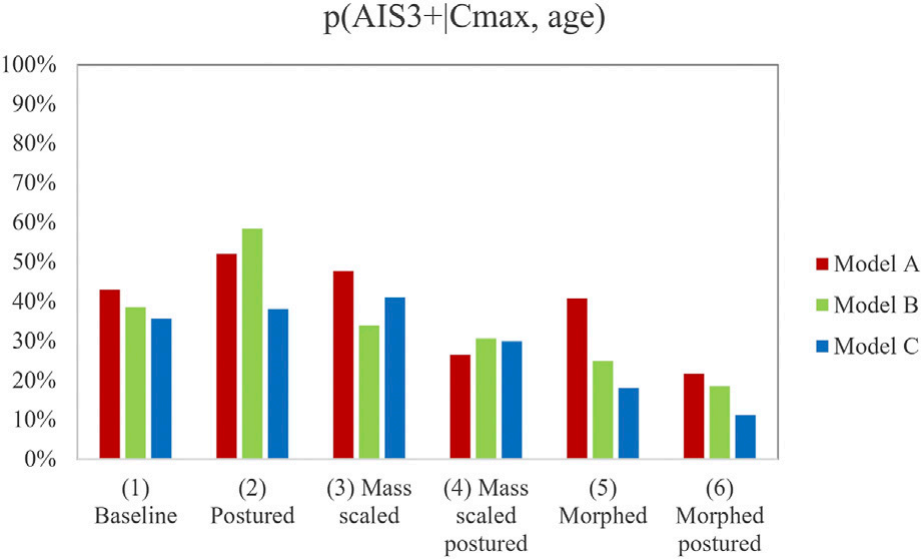
Probability of AIS3+ for the different model versions based on the Cmax metric. Note that there are 18 results to account for the differences in the position of the upper chest landmarks between PMHS A and B in the experiments.

The influence of the personalization of the model on the deformation criteria has been assessed for the diverse modifications of the HBM. Figure 4 and Figure 5 show the results obtained for the calculation of the probability of AIS3+ for the six model versions for each RS condition (PMHS A and B on RSv1 and PMHS C on RSv2). The summary of the results can be consulted in Supplementary Table 1-4.

In light of the results, the mass scaling and morphing of the HBM demonstrated a statistically significant influence on the prediction of AIS3+. However, mass-scaled and morphed models provided, in general, lower values of injury risk than the postured version (2), this last version being close to the reference PMHS sled tests that sustained 15, 5, and 10 fractured ribs respectively. Posturing did not show any statistically significant influence on injury prediction and showed a lower p (AIS3+) when it was combined with the other personalization techniques (versions 4 and 6). This could demonstrate that the separate personalization techniques do not lead to linear trends when they are used in combination. Additionally, those models in which the posturing was performed (2, 4, and 6) successfully predicted that the lower right chest landmark suffered the maximum deformation, while the non-postured model predicted that this deformation would happen at the upper left landmark (see Supplementary Figures 1-1–1-4).

It has to be noted that the results from the PMHS tests were used so that the HBM could be simulated under realistic impact conditions. It would be reasonable to expect that increased levels of personalization would result in improved predictions of risk. However, it is difficult to establish a direct comparison between the risk predicted by the HBM and the risk observed in the PMHS tests. First, even if the three experimental tests resulted in AIS3+ injuries, an exact calculation of the risk of AIS3+ injuries in the experimental loading conditions is not feasible with such a limited sample of PMHS tests. In addition, the definition of the AIS level based on rib fractures encompasses very different injury patterns (i.e. different numbers of rib fractures would result in the same AIS3 score depending on their location in the rib cage). This is why this study does not intend to highlight which personalization technique results in more biofidelic predictions, but rather how sensitive the injury criteria used with HBM are to the personalization of the models. From a more general perspective, if a criterion is not sensitive enough to change when the model is personalized, then there is not really a reason for not using the normalized, standard HBM.

This study is subject to some limitations. First, only three PMHS sled tests were taken as a reference for the comparison with the different model versions of the thoracic injury risk under oblique impact. Thus, more experimental data are required covering different impact angles, subject ages, sex, and anthropometrical characteristics for supporting the conclusions of this study. In terms of methodology, the morphing personalization technique was implemented attending to the external anthropometry, thus internal subject-specific geometry was not personalized. Since the scope of this study was to evaluate the influence of the subject-specific posture, mass, and anthropometry only, soft and hard tissue material properties remained unmodified in the SAFER HBM model. It should be noted that the anthropometry of PMHS A and PMHS B was averaged to develop a single personalized HBM. This was decided due to the large similarities in anthropometry and initial posture of the two subjects that would have resulted in minimal differences in the corresponding HBM.

## 5 Conclusion

This study analyzed the influence of different personalization techniques on the probability of chest injury predictions of HBM. Despite having led to statistically significant differences in the probability of AIS3+ calculations, mass-scaled and morphed model versions provided, in general, lower values of injury risk than the baseline model and the postured version (1 and 2). The postured model version (2) exhibited the highest values of probability of injury and resulted in predictions that closer resembled the injuries observed in the reference PMHS tests. Furthermore, the models in which the posturing was performed (2, 4, and 6) successfully predicted that the lower right chest landmark suffered the maximum deformation as occurred in the PMHS tests. Additionally, this study found that PC Score-based prediction showed higher values of p (AIS3+) than the prediction based on Cmax for the loading conditions and personalization techniques analyzed. Previous studies have suggested only minor differences between the two criteria, but the results included here suggest that these two criteria will result in significantly different predictions if the chest is loaded more asymmetrically.

## Data Availability

The original contributions presented in the study are included in the article/Supplementary Material, further inquiries can be directed to the corresponding authors.
