# Supplementary material for: Assessment of the sensitivity of thoracic injury criteria to subject-specific characteristics using human body models
Source: Front Bioeng Biotechnol. 2023 Feb 13;11:1106554. doi: 10.3389/fbioe.2023.1106554 (PMC9968747; doi:10.3389/fbioe.2023.1106554)
Supplement: Supplementary file 1 [file DataSheet1.docx]

Supplementary Material

Table 1-1: Subjects and baseline THUMS anthropometry expressed in mm. All dimensions were measured as specified in the NHTSA Data Reference Guide, Version 5, Volume II: Biomechanical Tests (May 2001).

|  | | RSv 1 | | RSv 2 |
| --- | --- | --- | --- | --- |
| ANTHROPOMETRIC MEASUREMENT | | Pmhs A | Pmhs B | Pmhs C |
| Seated Height-top of head to bottom of feet | | 963 | 1038 | 1040 |
| Seated Head to Buttock | | 753 | 802 | 832 |
| Seated Hip to Knee length | | 392 | 364 | 387 |
| Seated Chest Breadth | 4^th^ Rib | 256 | 277 | 284 |
|  | 8^th^ Rib | 260 | 270 | 289 |
| Seated Chest Depth | 4^th^ Rib | 240 | 202 | 201 |
|  | 8^th^ Rib | 250 | 202 | 202 |
| Seated Chest Circumference | 4^th^ Rib | 858 | 865 | 856 |
|  | 8^th^ Rib | 870 | 815 | 837 |
| Interscye | | 297 | 293 | 299 |
| Top of Head to T1 | | 201 | 202 | 273 |
| Waist Depth-Umbilicus | | 170 | 135 | 152 |
| Waist Breadth | | 263 | 257 | 302 |
| Shoulder Breadth (Biacromial) | | 331 | 357 | 354 |
| Hip Breadth | | 292 | 292 | 319 |
| Buttock Depth | | 167 | 155 | 178 |
| Shoulder to Elbow | | 319 | 305 | 357 |
| Forearm to Hand | | 248 | 235 | 243 |
| Foot Breadth | | 70 | 68 | 68 |
| Foot Length | | 247 | 215 | 237 |
| Head Length | | 202 | 197 | 218 |
| Head Breadth | | 140 | 147 | 135 |
| Head Height | | 208 | 195 | 219 |
| Head Circumference | | 553 | 553 | 616 |
| Neck Circumference | | 334 | 360 | 347 |
| Waist Circumference (Umbilicus) | | 702 | 706 | 816 |
| Buttock Circumference | | 776 | 722 | 830 |
| Thigh Circumference | | 321 | 355 | 391 |
| Lower Thigh Circumference | | 259 | 322 | 340 |
| Knee Circumference | | 342 | 340 | 352 |
| Calf Circumference | | 240 | 244 | 284 |
| Ankle Circumference | | 242 | 212 | 202 |
| Scye (Armpit) Circumference | | 235 | 253 | 251 |
| Bicep Circumference | | 195 | 218 | 222 |
| Elbow Circumference | | 222 | 233 | 239 |
| Forearm Circumference | | 150 | 207 | 197 |
| Wrist Circumference | | 159 | 159 | 151 |
| Weight (kg) | | 47 | 53 | 57 |

Table 1-2: Angle formed with the horizontal of the selected landmarks in the spine for RSv1 (degree).

| *Subject* | Pmhs A | Pmhs B | Baseline | Personalized |
| --- | --- | --- | --- | --- |
| *α _Head-T1_* | 66 | 52 | 95 | 66 |
| *α _T1-T8_* | 63 | 76 | 81 | 76 |
| *α _T8-L2_* | 120 | 99 | 112 | 120 |
| *α _L2-H-Point_* | 147 | 142 | 141 | 140 |

Table 1-3: Angle formed with the horizontal of the selected landmarks in the spine for RSv2 (degree).

| *Subject* | Pmhs C | Baseline | Personalized |
| --- | --- | --- | --- |
| *α _Head-T4_* | 54 | 89 | 63 |
| *α _T4-T7_* | 76 | 85 | 79 |
| *α _T7-L1_* | 124 | 107 | 121 |
| *α _L1-H-Point_* | 135 | 139 | 139 |

Figure 1-1: Maximum deflection of the PMHS’ rib landmarks.


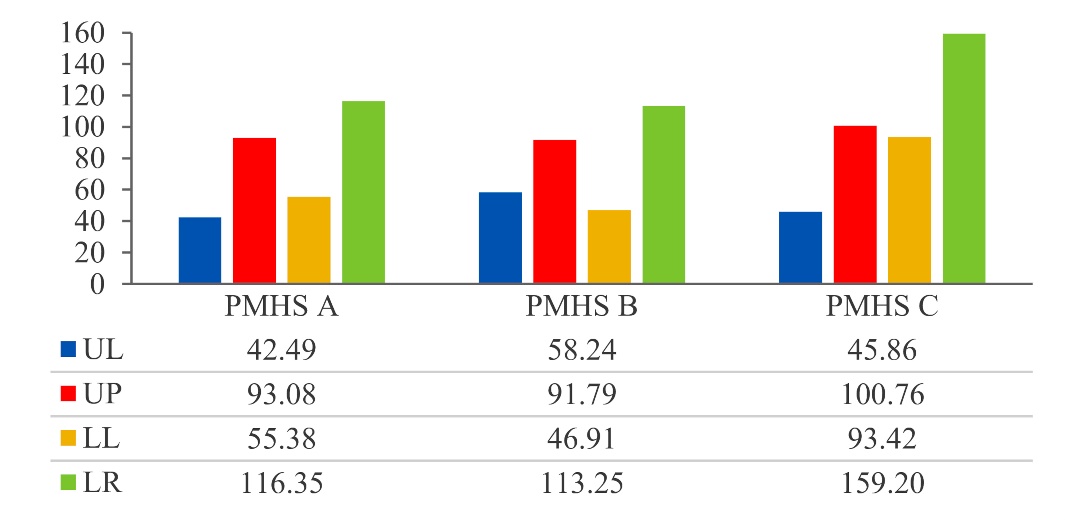


Figure 1-2: Maximum deflection of the rib landmarks corresponding to the A model versions.


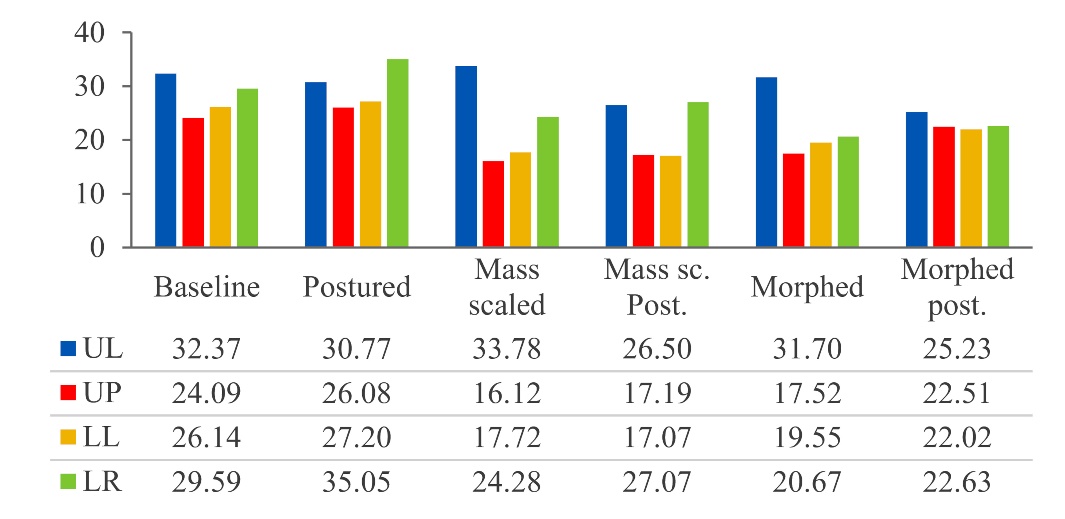


Figure 1-3: Maximum deflection of the rib landmarks corresponding to the B model versions


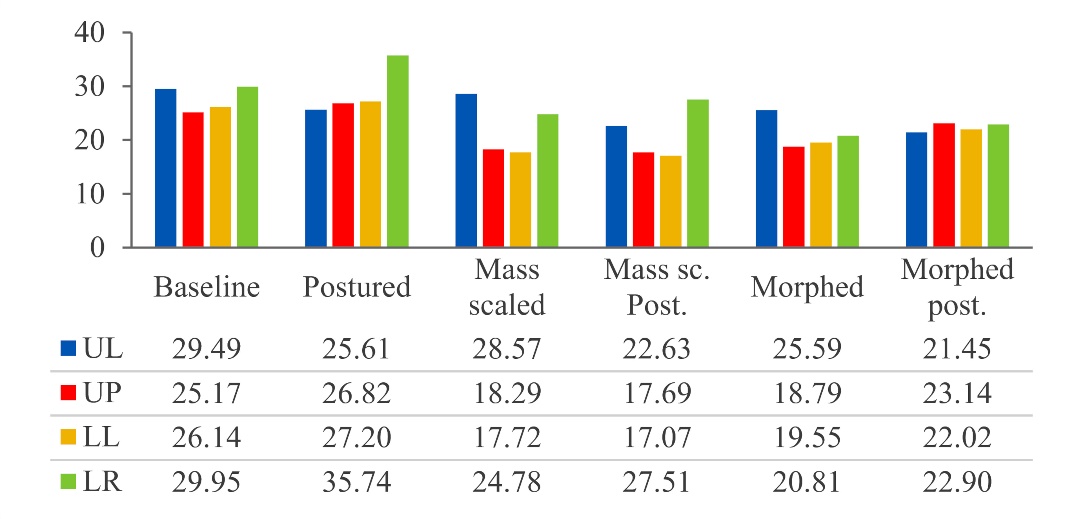


Figure 1-4: Maximum deflection of the rib landmarks corresponding to the C model versions


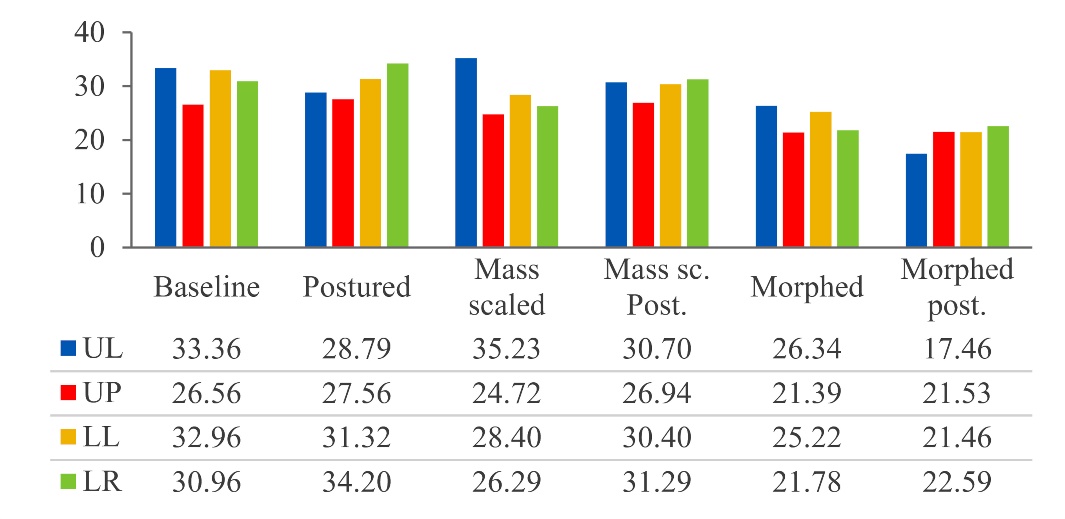


Table 1-4. Simulation results including deflection values and injury metrics for P(AIS3+) calculation.

| RSv | Model | Version | Age (y.o.) | AIS3+ | UL (mm) | UR (mm) | LL (mm) | LR (mm) | Updif (mm) | LOWdif (mm) | Uptot (mm) | LOWtot (mm) | Cmax | P(AIS3+\| Cmax, age) | PC score | P(AIS3+\| PC score, age) |
| --- | --- | --- | --- | --- | --- | --- | --- | --- | --- | --- | --- | --- | --- | --- | --- | --- |
| RSv1 | A | 1-Bas | 66 | Y | 32.4 | 24.1 | 26.1 | 29.6 | 24.5 | 18.9 | 56.5 | 55.7 | 32.4 | 43.00% | 5.49 | 66.83% |
| RSv1 | A | 2-Pos | 66 | Y | 30.8 | 26.1 | 27.2 | 35.0 | 29.0 | 18.7 | 56.9 | 62.2 | 35.0 | 51.99% | 5.94 | 76.08% |
| RSv1 | A | 3-Mass | 66 | Y | 33.8 | 16.1 | 17.7 | 24.3 | 22.1 | 16.1 | 49.9 | 42.0 | 33.8 | 47.70% | 4.60 | 45.97% |
| RSv1 | A | 4-MassPos | 66 | Y | 26.5 | 17.2 | 17.1 | 27.1 | 16.7 | 18.2 | 43.7 | 44.1 | 27.1 | 26.55% | 4.31 | 39.16% |
| RSv1 | A | 5-Morph | 66 | Y | 31.7 | 17.5 | 19.6 | 20.7 | 25.4 | 13.6 | 49.2 | 40.2 | 31.7 | 40.79% | 4.59 | 45.59% |
| RSv1 | A | 6-MorPos | 66 | Y | 25.2 | 22.5 | 22.0 | 22.6 | 19.3 | 13.0 | 47.7 | 44.6 | 25.2 | 21.61% | 4.36 | 40.15% |
| RSv1 | B | 1-Bas | 68 | Y | 29.5 | 25.2 | 26.1 | 30.0 | 13.0 | 19.4 | 54.7 | 56.1 | 30.0 | 38.48% | 4.88 | 56.99% |
| RSv1 | B | 2-Pos | 68 | Y | 25.6 | 26.8 | 27.2 | 35.7 | 14.5 | 19.1 | 52.4 | 62.9 | 35.7 | 58.48% | 5.11 | 62.55% |
| RSv1 | B | 3-Mass | 68 | Y | 28.6 | 18.3 | 17.7 | 24.8 | 12.7 | 16.6 | 46.9 | 42.5 | 28.6 | 33.93% | 4.07 | 37.07% |
| RSv1 | B | 4-MassPos | 68 | Y | 22.6 | 17.7 | 17.1 | 27.5 | 6.8 | 18.6 | 40.3 | 44.6 | 27.5 | 30.60% | 3.75 | 29.68% |
| RSv1 | B | 5-Morph | 68 | Y | 25.6 | 18.8 | 19.6 | 20.8 | 13.9 | 14.0 | 44.4 | 40.4 | 25.6 | 24.91% | 3.89 | 32.77% |
| RSv1 | B | 6-MorPos | 68 | Y | 21.4 | 23.1 | 22.0 | 22.9 | 7.9 | 13.3 | 44.6 | 44.9 | 23.1 | 18.48% | 3.71 | 28.80% |
| RSv2 | C | 1-Bas | 60 | Y | 33.4 | 26.6 | 33.0 | 31.0 | 16.1 | 17.8 | 59.9 | 63.9 | 33.4 | 35.64% | 5.38 | 51.39% |
| RSv2 | C | 2-Pos | 60 | Y | 28.8 | 27.6 | 31.3 | 34.2 | 9.4 | 26.6 | 56.4 | 65.5 | 34.2 | 38.08% | 5.36 | 50.96% |
| RSv2 | C | 3-Mass | 60 | Y | 35.2 | 24.7 | 28.4 | 26.3 | 13.5 | 17.1 | 59.9 | 54.7 | 35.2 | 41.08% | 4.91 | 41.25% |
| RSv2 | C | 4-MassPos | 60 | Y | 30.7 | 26.9 | 30.4 | 31.3 | 6.0 | 21.0 | 57.6 | 61.7 | 31.3 | 29.92% | 4.86 | 40.17% |
| RSv2 | C | 5-Morph | 60 | Y | 26.3 | 21.4 | 25.2 | 21.8 | 11.7 | 5.0 | 47.7 | 47.0 | 26.3 | 18.09% | 3.71 | 18.93% |
| RSv2 | C | 6-MorPos | 60 | Y | 17.5 | 21.5 | 21.5 | 22.6 | 7.9 | 16.5 | 39.0 | 44.0 | 22.6 | 11.23% | 3.66 | 18.24% |
